# The Office of Health Assessment and Translation: A Problem-Solving Resource for the National Toxicology Program

**DOI:** 10.1289/ehp.1103645

**Published:** 2011-05

**Authors:** John R. Bucher, Kristina Thayer, Linda S. Birnbaum

**Affiliations:** National Institute of Environmental Health Sciences, National Institutes of Health, Department of Health and Human Services, Research Triangle Park, North Carolina, E-mail: bucher@niehs.nih.gov

The National Toxicology Program (NTP) Center for the Evaluation of Risks to Human Reproduction (CERHR) was established in 1998. CERHR served as an environmental health resource providing in-depth scientific assessments of effects on reproduction and development caused by agents to which humans are exposed. To our knowledge, CERHR was the only resource of its kind, producing evaluations that considered toxicity findings in the context of current human exposures to derive “level-of-concern” conclusions. This qualitative integration step is what distinguished CERHR documents from more traditional hazard evaluations prepared by other agencies.

When CERHR was established, the focus on reproduction and development was appropriate because of a strong interest in these health outcomes by the public, regulatory and health agencies, and the scientific community. In addition, a rationale for creating CERHR was the sense of a lack of uniformity across state and federal agencies in interpreting experimental animal studies of reproduction and development. CERHR was envisioned as a mechanism to apply a consistent strategy for interpreting these data. Although this need remains, we believe that the approaches used for CERHR evaluations should also be extended to other important health outcomes. Many chemicals display more than one type of toxicity, that is, carcinogens are often immunotoxicants, and reproductive and developmental toxicants may influence many endocrine-sensitive systems. A strict focus on reproductive and developmental end points evaluated in the context of current human exposures may not result in the most health protective levels of concern, and could be confusing to the public. From a public health perspective, understanding the implications of current human exposures should include consideration of all relevant health effects. Also, the NTP and the broader toxicology community need to confront the challenging scientific questions involved in utilizing information from the Toxicology in the 21st Century initiative (Collins et al. 2008). To do this we need a mechanism to systematically explore linkages between “toxicity pathways” and disease outcomes. To provide this, CERHR has spent the last 2 years in transition, laying the groundwork to become a more flexible scientific analysis program, while continuing to be grounded and recognized as a unique and important public health resource for the interpretation of reproductive and developmental hazards to humans.

This evolution of CERHR is a response to the changing and increasing demands on both the NTP analysis and research programs. ”What does it mean?” is a question we increasingly want to answer, as our research and testing tools become more sophisticated and mechanistically based. A change in CERHR’s scope will also bring its work more in line with two recent initiatives established within the NTP that have mandates to address a broad range of health effects (Bucher 2008). In 2007 the NTP established a biomolecular screening program to administer its High Throughput Screening (HTS) Initiative in collaboration with our Tox21 partners (Schmidt 2009). This program takes advantage of technological advances in molecular biology and computer science to screen for mechanistic targets or “toxicity pathways” considered critical to adverse health effects. The host susceptibility program was also established in 2007 to study the genetic basis for differences in susceptibility that may lead to a better understanding of how substances in our environment may be hazardous to some individuals and not to others.

On 11–13 January 2011, CERHR launched its expanded role by convening a diverse group of experts in toxicology, epidemiology, bioinformatics, and endocrinology to assess the strength of the literature linking selected environmental agents and exposures with diabetes and obesity (NTP 2011). Consideration was given to an array of information ranging from epidemiological findings and experimental animal and mechanistic data to screens of toxicity and disease pathways using HTS and literature curation methodologies. The use of several new analysis tools revealed novel linkages between a number of environmental agents and obesity or diabetes. These exciting findings are now being collated for publication.

To fulfill its mission, the NTP is developing more innovative and flexible approaches for information and data integration, both across different programs within the NTP and across the different types of data that are generated and utilized (i.e., mechanistic or high throughput; “hypothesis-driven” animal studies of the type undertaken by National Institute of Environmental Health Sciences (NIEHS)-funded extramural grantees; and toxicology studies conducted for the purpose of safety assessment). Recent experience with bisphenol A highlights the public’s confusion and the waste of scientific resources that can occur when these different types of scientific literature are developed on parallel, but separate, paths (Bucher 2009). The evolution of CERHR is an important part of this information integration effort, and CERHR’s new role calls for a new name: the Office of Health Assessment and Translation. Under the leadership of Kristina Thayer, the Office of Health Assessment and Translation will be the NTP focal point for the thoughtful and deliberative integration of relevant information of all types in health assessments for the protection of public health.

## Figures and Tables

**Figure:**
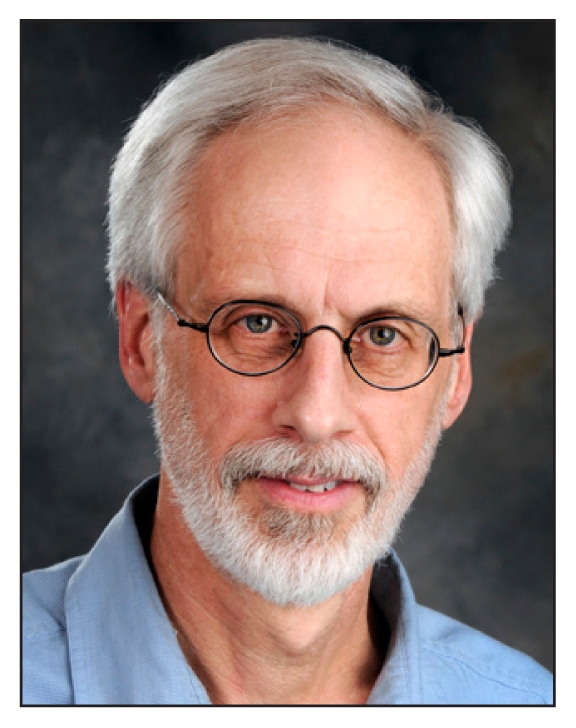
John R. Bucher

**Figure:**
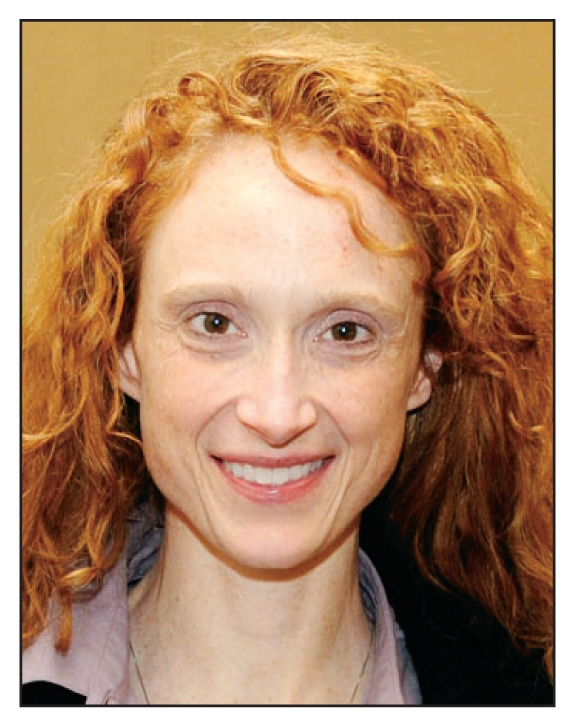
Kristina Thayer

**Figure:**
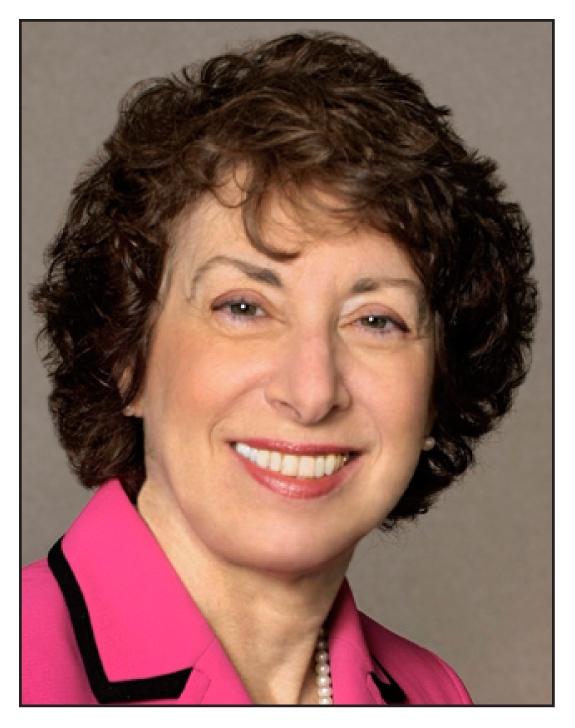
Linda S. Birnbaum
